# 5-Hydr­oxy-1,7-bis­(1*H*-indol-3-yl)hepta-1,4,6-trien-3-one hemihydrate

**DOI:** 10.1107/S1600536809018571

**Published:** 2009-05-29

**Authors:** Jun-Hai Li, Gang-Chun Sun, Joel T. Mague, Jing-Xi Xie

**Affiliations:** a School of Chemistry and Chemical Engineering, Henan University of Technology, Zhengzhou 450001, People’s Republic of China; b Department of Chemistry, Tulane University, New Orleans, LA 70118, USA

## Abstract

The title compound, C_23_H_18_N_2_O_2_·0.5H_2_O, a derivative of the biologically active compound curcumin, crystallizes with two organic mol­ecules and a solvent water mol­ecule in the asymmetric unit. Each of the two independent mol­ecules is close to being planar (the dihedral angles between the indole ring systems are approximately 9 and 12°) and each exists in the keto–enol form. There is an intra­molecular O—H⋯O hydrogen bond between the keto and enol groups. In the crystal, the components interact by way of N—H⋯N, N—H⋯O and O—H⋯O hydrogen bonds.

## Related literature

For biological activities of curcumin and related analogues, see: Ammon & Wahl (1991 ▶); Lee (2004 ▶). For related structures, see: Arrieta *et al.* (2000 ▶); Mague *et al.* (2004 ▶); Pedersen *et al.* (1985 ▶).
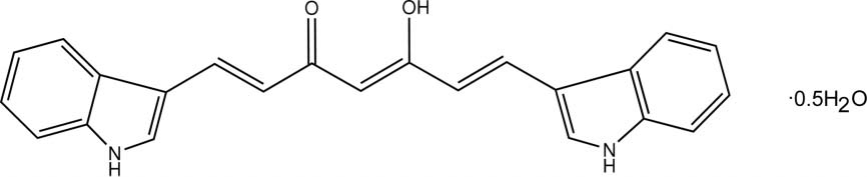


## Experimental

### 

#### Crystal data

C_23_H_18_N_2_O_2_·0.5H_2_O
*M* *_r_* = 363.4
Orthorhombic, 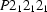

*a* = 5.5285 (7) Å
*b* = 22.916 (3) Å
*c* = 28.194 (4) Å
*V* = 3571.9 (8) Å^3^
*Z* = 8
Mo *K*α radiation
μ = 0.09 mm^−1^
*T* = 100 K
0.29 × 0.20 × 0.18 mm

#### Data collection

Bruker APEX-I CCD diffractometer
Absorption correction: multi-scan (*SADABS*; Sheldrick, 2008*b* ▶) *T*_min_ = 0.913, *T*_max_ = 0.984
61988 measured reflections
5073 independent reflections
4650 reflections with *I* > 2σ(*I*)
*R*_int_ = 0.038

#### Refinement

*R*[*F* ^2^ > 2σ(*F* ^2^)] = 0.033
*wR*(*F* ^2^) = 0.084
*S* = 1.06
5073 reflections
496 parameters
H-atom parameters constrained
Δρ_max_ = 0.19 e Å^−3^
Δρ_min_ = −0.15 e Å^−3^
Absolute structure: the absolute structure could not be determined with certainty

### 

Data collection: *APEX2* (Bruker, 2009 ▶); cell refinement: *SAINT* (Bruker 2008 ▶); data reduction: *SAINT*; program(s) used to solve structure: *SHELXS97* (Sheldrick, 2008*a* ▶); program(s) used to refine structure: *SHELXL97* (Sheldrick, 2008*a* ▶); molecular graphics: *SHELXTL* (Sheldrick, 2008*a* ▶); software used to prepare material for publication: *SHELXTL*.

## Supplementary Material

Crystal structure: contains datablocks I, global. DOI: 10.1107/S1600536809018571/bx2202sup1.cif

Structure factors: contains datablocks I. DOI: 10.1107/S1600536809018571/bx2202Isup2.hkl

Additional supplementary materials:  crystallographic information; 3D view; checkCIF report

## Figures and Tables

**Table 1:** Hydrogen-bond geometry (Å, °)

*D*—H⋯*A*	*D*—H	H⋯*A*	*D*⋯*A*	*D*—H⋯*A*
O1—H1O⋯O2	0.98	1.58	2.5148 (18)	158
N1—H1N⋯N1^i^	0.90	2.55	3.4254 (18)	167
N2—H2N⋯O5^ii^	0.86	2.08	2.912 (2)	161
O3—H3O⋯O4	0.90	1.71	2.5120 (19)	147
O5—H5O*A*⋯O5^iii^	0.90	2.11	3.0062 (10)	177
O5—H5O*B*⋯O4^iv^	0.91	1.83	2.7128 (18)	162

Symmetry codes: (i) 
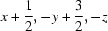
; (ii) 
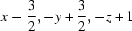
; (iii) 
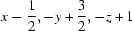
; (iv) 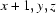.

**Fig. 1.**
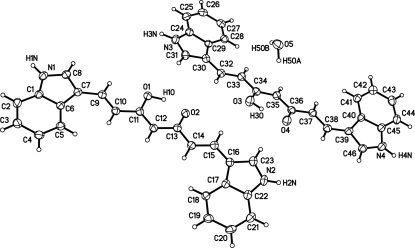
The molecular structure of (I). Displacement ellipsoids are drawn at the 50% probability level and H-atoms are represented by spheres of arbitrary radius.

**Fig. 2.**
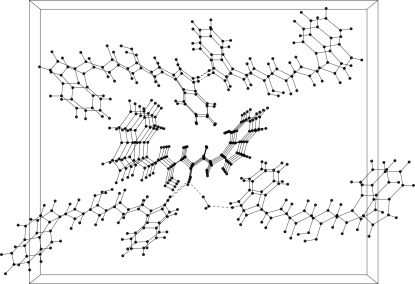
The cystal packing of (I), viewed down the a axis.

## supplementary crystallographic information

### Comment

Curcumin and related analogues have antioxidant, antibacterial, anti-inflammatory and other biological activities (Ammon & Wahl, 1991; Lee, 2004). This fact has made these molecules, as well as synthetic analogues (Pedersen *et al.*, 1985), interesting targets for structural study(Arrieta *et al.*, 2000; Mague *et al.*, 2004).

The title compound, (I) (Fig. 1), is new to this class of compounds and crystallizes with two molecules and a solvent water molecule in the asymmetric unit.The two independent molecules are close to being planar as indicated by the torsion angles along the chains with molecule 2 being less planar than molecule 1. This is also shown by the dihedral angles between the indolyl moieties and the central C_3_O_2_ unit which are 3.06 (8) and 9.09 (9) ° in molecule 1 and 9.90 (9) and 21.07 (9) ° in molecule 2. The molecular geometry and H-atom locations reveal that 3,5-dione moieties of both molecules exist in the keto-enol form and display intramolecular C—H···O hydrogen bonds between the keto and enol groups.

The packing of the molecules in the lattice is also stabilized by by strong O—H···O and N—H···O intermolecular hydrogen bonds.

### Experimental

2,4-Pentanedione (2.0 g, 20 mmol) and boric anhydride (1.0 g, 15 mmol) were dissolved in 20 ml of EtOAc.The solution was stirred at 60 °C for 4 h,then 3-indolylaldehyde (5.8 g, 40 mmol) dissolved in 60 ml of EtOAc and tributyl borate (9.2 g, 40 mmol) were added. After stirring for 30 min at a temperature of 85 °C, n-butylamine (3.0 mL, 30 mmol) dissolved in 10 ml of EtOAc was added dropwise over 30 min and the stirring was continued for 5 h at 85 °C. The mixture was kept in 5 °C overnight and then hydrolyzed by adding 30 ml of 1 N HCl and stirring for 2 h at 60 °C. The organic layer was separated and the aqueous layer was extracted with EtOAc. The combined organic layers were washed until neutral and dried over anhydrous sodium sulfate. The solvent was removed *in vacuo*, and the crude product was purified by recrystallization from MeOH to give 8.1 g of red crystalline solid in 59% yield. mp 214–216 °C. Crystals suitable for X-ray determination were obtained by slowly evaporating an acetonitrile solution at room temperature over 2 days.

### Refinement

All Hydrogen atoms were found in difference maps.Those attached to carbon were placed in calculated positions (C—H = 0.95%A) while those attached to nitrogen and the lattice water were placed in locations derived from the difference map. All H atoms were included as riding contributions with isotropic displacement parameters 1.2 times those of the attached atoms.

### Crystal data

**Table:**

C_23_H_18_N_2_O_2_·0.5H_2_O	*F*(000) = 1528
*M**_r_* = 363.4	*D*_x_ = 1.352 Mg m^−^^3^
Orthorhombic, *P*2_1_2_1_2_1_	Mo *K*α radiation, λ = 0.71073 Å
Hall symbol: P 2ac 2ab	Cell parameters from 9666 reflections
*a* = 5.5285 (7) Å	θ = 2.3–28.3°
*b* = 22.916 (3) Å	µ = 0.09 mm^−^^1^
*c* = 28.194 (4) Å	*T* = 100 K
*V* = 3571.9 (8) Å^3^	Block, red
*Z* = 8	0.29 × 0.20 × 0.18 mm

### Data collection

**Table:**

Bruker APEX-I CCD diffractometer	5073 independent reflections
Radiation source: fine-focus sealed tube	4650 reflections with *I* > 2σ(*I*)
graphite	*R*_int_ = 0.038
φ and ω scans	θ_max_ = 28.3°, θ_min_ = 2.3°
Absorption correction: multi-scan (*SADABS*; Sheldrick, 2008b)	*h* = −7→7
*T*_min_ = 0.913, *T*_max_ = 0.984	*k* = −30→30
61988 measured reflections	*l* = −37→37

### Refinement

**Table:**

Refinement on *F*^2^	Secondary atom site location: difference Fourier map
Least-squares matrix: full	Hydrogen site location: inferred from neighbouring sites
*R*[*F*^2^ > 2σ(*F*^2^)] = 0.033	H-atom parameters constrained
*wR*(*F*^2^) = 0.084	*w* = 1/[σ^2^(*F*_o_^2^) + (0.0406*P*)^2^ + 0.8427*P*] where *P* = (*F*_o_^2^ + 2*F*_c_^2^)/3
*S* = 1.06	(Δ/σ)_max_ < 0.001
5073 reflections	Δρ_max_ = 0.19 e Å^−^^3^
496 parameters	Δρ_min_ = −0.15 e Å^−^^3^
0 restraints	Absolute structure: The absolute structure could not be determined with certainty
Primary atom site location: structure-invariant direct methods	

### Special details

**Table:**

Experimental. The diffraction data were obtained from 3 sets of 400 frames, each of width 0.5 °. in omega, colllected at phi = 0.00, 90.00 and 180.00 °. and 2 sets of 800 frames, each of width 0.45 ° in phi, collected at omega = -30.00 and 210.00 °. The scan time was 20 sec/frame.
Geometry. All e.s.d.'s (except the e.s.d. in the dihedral angle between two l.s. planes) are estimated using the full covariance matrix. The cell e.s.d.'s are taken into account individually in the estimation of e.s.d.'s in distances, angles and torsion angles; correlations between e.s.d.'s in cell parameters are only used when they are defined by crystal symmetry. An approximate (isotropic) treatment of cell e.s.d.'s is used for estimating e.s.d.'s involving l.s. planes.
Refinement. Refinement of *F*^2^ against ALL reflections. The weighted *R*-factor *wR* and goodness of fit *S* are based on *F*^2^, conventional *R*-factors *R* are based on *F*, with *F* set to zero for negative *F*^2^. The threshold expression of *F*^2^ > σ(*F*^2^) is used only for calculating *R*-factors(gt) *etc*. and is not relevant to the choice of reflections for refinement. *R*-factors based on *F*^2^ are statistically about twice as large as those based on *F*, and *R*- factors based on ALL data will be even larger. H-atoms attached to carbon were placed in calculated positions (C—H = 0.95%A) while those attached to nitrogen and oxygen were placed in locations derived from a difference map. All H atoms were included as riding contributions with isotropic displacement parameters 1.2 times those of the attached atoms.

### Fractional atomic coordinates and isotropic or equivalent isotropic displacement parameters (Å^2^)

**Table:**

	*x*	*y*	*z*	*U*_iso_*/*U*_eq_	
O1	0.5647 (2)	0.68150 (5)	0.20031 (4)	0.0263 (3)	
H1O	0.4628	0.6733	0.2280	0.032*	
O2	0.2393 (2)	0.67798 (5)	0.26257 (4)	0.0270 (3)	
N1	1.2107 (3)	0.77241 (7)	0.03091 (5)	0.0282 (3)	
H1N	1.3528	0.7664	0.0164	0.034*	
N2	−0.6358 (3)	0.77437 (7)	0.39136 (5)	0.0282 (3)	
H2N	−0.7177	0.7655	0.4164	0.034*	
C1	1.0616 (3)	0.81969 (7)	0.02378 (6)	0.0243 (3)	
C2	1.0791 (4)	0.86367 (8)	−0.01049 (6)	0.0306 (4)	
H2	1.2088	0.8646	−0.0326	0.037*	
C3	0.9002 (4)	0.90557 (9)	−0.01081 (7)	0.0335 (4)	
H3	0.9065	0.9361	−0.0335	0.040*	
C4	0.7087 (4)	0.90380 (8)	0.02193 (7)	0.0311 (4)	
H4	0.5877	0.9332	0.0209	0.037*	
C5	0.6925 (3)	0.86010 (8)	0.05568 (6)	0.0262 (4)	
H5	0.5625	0.8596	0.0777	0.031*	
C6	0.8706 (3)	0.81654 (7)	0.05694 (5)	0.0220 (3)	
C7	0.9142 (3)	0.76498 (7)	0.08545 (6)	0.0226 (3)	
C8	1.1237 (3)	0.73994 (8)	0.06790 (6)	0.0260 (4)	
H8	1.1964	0.7053	0.0798	0.031*	
C9	0.7844 (3)	0.74040 (7)	0.12523 (6)	0.0232 (3)	
H9	0.8520	0.7061	0.1388	0.028*	
C10	0.5801 (3)	0.76008 (7)	0.14533 (6)	0.0233 (3)	
H10	0.5078	0.7945	0.1330	0.028*	
C11	0.4647 (3)	0.73097 (7)	0.18516 (6)	0.0220 (3)	
C12	0.2620 (3)	0.75363 (7)	0.20656 (6)	0.0240 (3)	
H12	0.1911	0.7882	0.1942	0.029*	
C13	0.1558 (3)	0.72601 (7)	0.24710 (6)	0.0228 (3)	
C14	−0.0496 (3)	0.75494 (7)	0.26938 (6)	0.0240 (3)	
H14	−0.1215	0.7870	0.2534	0.029*	
C15	−0.1434 (3)	0.73862 (7)	0.31154 (6)	0.0237 (3)	
H15	−0.0676	0.7066	0.3270	0.028*	
C16	−0.3456 (3)	0.76457 (7)	0.33547 (6)	0.0234 (3)	
C17	−0.4999 (3)	0.81296 (7)	0.32258 (6)	0.0222 (3)	
C18	−0.5089 (3)	0.85307 (7)	0.28474 (6)	0.0247 (3)	
H18	−0.3885	0.8524	0.2607	0.030*	
C19	−0.6946 (3)	0.89340 (8)	0.28295 (6)	0.0283 (4)	
H19	−0.7012	0.9202	0.2573	0.034*	
C20	−0.8737 (3)	0.89564 (8)	0.31818 (6)	0.0292 (4)	
H20	−1.0004	0.9235	0.3158	0.035*	
C21	−0.8678 (3)	0.85780 (8)	0.35632 (6)	0.0281 (4)	
H21	−0.9871	0.8594	0.3805	0.034*	
C22	−0.6801 (3)	0.81715 (7)	0.35796 (6)	0.0245 (3)	
C23	−0.4386 (4)	0.74313 (8)	0.37779 (6)	0.0272 (4)	
H23	−0.3734	0.7111	0.3949	0.033*	
O3	0.1070 (3)	0.64304 (5)	0.39766 (5)	0.0326 (3)	
H3O	−0.0043	0.6575	0.4177	0.039*	
O4	−0.2323 (3)	0.64271 (5)	0.45693 (5)	0.0307 (3)	
N3	0.9824 (3)	0.55581 (7)	0.27354 (5)	0.0306 (3)	
H3N	1.1011	0.5666	0.2516	0.037*	
N4	−0.9549 (3)	0.54556 (7)	0.61481 (6)	0.0310 (3)	
H4N	−1.0834	0.5551	0.6333	0.037*	
C24	0.9811 (3)	0.50219 (8)	0.29622 (6)	0.0257 (4)	
C25	1.1444 (4)	0.45637 (8)	0.29259 (7)	0.0303 (4)	
H25	1.2788	0.4582	0.2716	0.036*	
C26	1.1037 (4)	0.40804 (8)	0.32061 (6)	0.0307 (4)	
H26	1.2131	0.3761	0.3194	0.037*	
C27	0.9031 (4)	0.40543 (8)	0.35091 (6)	0.0291 (4)	
H27	0.8779	0.3714	0.3695	0.035*	
C28	0.7414 (3)	0.45103 (7)	0.35440 (6)	0.0261 (4)	
H28	0.6063	0.4485	0.3751	0.031*	
C29	0.7792 (3)	0.50139 (7)	0.32683 (6)	0.0224 (3)	
C30	0.6606 (3)	0.55752 (7)	0.32232 (6)	0.0243 (3)	
C31	0.7936 (4)	0.58820 (8)	0.28923 (6)	0.0281 (4)	
H31	0.7577	0.6267	0.2789	0.034*	
C32	0.4623 (3)	0.58270 (7)	0.34775 (6)	0.0247 (3)	
H32	0.4202	0.6216	0.3397	0.030*	
C33	0.3288 (3)	0.55712 (7)	0.38193 (6)	0.0250 (3)	
H33	0.3622	0.5176	0.3899	0.030*	
C34	0.1383 (3)	0.58676 (7)	0.40696 (6)	0.0239 (3)	
C35	−0.0057 (3)	0.55879 (7)	0.44003 (6)	0.0250 (3)	
H35	0.0204	0.5185	0.4462	0.030*	
C36	−0.1906 (3)	0.58855 (7)	0.46478 (6)	0.0243 (3)	
C37	−0.3279 (3)	0.55676 (7)	0.50033 (6)	0.0247 (3)	
H37	−0.2769	0.5185	0.5087	0.030*	
C38	−0.5247 (3)	0.57924 (7)	0.52196 (6)	0.0253 (3)	
H38	−0.5797	0.6160	0.5108	0.030*	
C39	−0.6598 (3)	0.55357 (7)	0.56006 (6)	0.0243 (3)	
C40	−0.6107 (3)	0.50315 (7)	0.58930 (6)	0.0237 (3)	
C41	−0.4243 (4)	0.46185 (8)	0.59151 (6)	0.0271 (4)	
H41	−0.2928	0.4637	0.5699	0.032*	
C42	−0.4335 (4)	0.41834 (8)	0.62545 (6)	0.0307 (4)	
H42	−0.3050	0.3909	0.6276	0.037*	
C43	−0.6307 (4)	0.41406 (8)	0.65692 (6)	0.0334 (4)	
H43	−0.6368	0.3827	0.6789	0.040*	
C44	−0.8153 (4)	0.45465 (8)	0.65628 (6)	0.0324 (4)	
H44	−0.9470	0.4523	0.6778	0.039*	
C45	−0.8006 (3)	0.49921 (8)	0.62277 (6)	0.0267 (4)	
C46	−0.8710 (3)	0.57775 (8)	0.57792 (6)	0.0278 (4)	
H46	−0.9463	0.6120	0.5660	0.033*	
O5	0.6429 (3)	0.73209 (6)	0.51507 (4)	0.0335 (3)	
H5OA	0.4914	0.7418	0.5068	0.040*	
H5OB	0.6609	0.6979	0.4989	0.040*	

### Atomic displacement parameters (Å^2^)

**Table:**

	*U*^11^	*U*^22^	*U*^33^	*U*^12^	*U*^13^	*U*^23^
O1	0.0264 (6)	0.0262 (6)	0.0264 (6)	0.0027 (5)	0.0007 (5)	0.0030 (5)
O2	0.0282 (6)	0.0263 (6)	0.0264 (6)	0.0006 (5)	0.0016 (5)	0.0037 (5)
N1	0.0231 (7)	0.0327 (8)	0.0288 (7)	0.0015 (7)	0.0048 (7)	−0.0026 (6)
N2	0.0298 (8)	0.0324 (7)	0.0223 (7)	−0.0052 (7)	0.0076 (6)	0.0000 (6)
C1	0.0215 (8)	0.0282 (8)	0.0233 (7)	−0.0026 (7)	−0.0001 (7)	−0.0029 (7)
C2	0.0264 (9)	0.0378 (10)	0.0278 (8)	−0.0071 (8)	0.0002 (8)	0.0033 (8)
C3	0.0289 (10)	0.0378 (10)	0.0339 (9)	−0.0048 (9)	−0.0039 (8)	0.0114 (8)
C4	0.0250 (9)	0.0307 (9)	0.0375 (9)	0.0011 (8)	−0.0040 (8)	0.0063 (8)
C5	0.0229 (8)	0.0287 (8)	0.0270 (8)	−0.0003 (7)	−0.0010 (7)	−0.0005 (7)
C6	0.0207 (8)	0.0253 (7)	0.0200 (7)	−0.0019 (7)	−0.0012 (6)	−0.0034 (6)
C7	0.0217 (8)	0.0239 (7)	0.0222 (7)	0.0000 (7)	−0.0004 (7)	−0.0032 (6)
C8	0.0245 (8)	0.0254 (8)	0.0280 (8)	0.0006 (7)	0.0009 (7)	−0.0020 (7)
C9	0.0235 (8)	0.0229 (7)	0.0233 (7)	−0.0015 (7)	−0.0019 (7)	−0.0019 (6)
C10	0.0235 (8)	0.0238 (7)	0.0227 (7)	0.0000 (7)	−0.0010 (7)	−0.0011 (6)
C11	0.0231 (8)	0.0225 (7)	0.0205 (7)	−0.0028 (7)	−0.0023 (7)	−0.0016 (6)
C12	0.0250 (8)	0.0237 (8)	0.0234 (8)	0.0011 (7)	0.0005 (7)	0.0015 (6)
C13	0.0230 (8)	0.0237 (7)	0.0218 (7)	−0.0031 (7)	−0.0019 (7)	−0.0024 (6)
C14	0.0243 (8)	0.0236 (7)	0.0242 (8)	0.0000 (7)	−0.0003 (7)	0.0001 (6)
C15	0.0250 (8)	0.0232 (7)	0.0230 (7)	−0.0026 (7)	−0.0009 (7)	−0.0018 (6)
C16	0.0260 (8)	0.0232 (7)	0.0210 (7)	−0.0028 (7)	0.0002 (7)	−0.0020 (6)
C17	0.0206 (8)	0.0247 (7)	0.0213 (7)	−0.0035 (7)	0.0000 (7)	−0.0044 (6)
C18	0.0223 (8)	0.0294 (8)	0.0226 (7)	−0.0007 (7)	0.0001 (7)	−0.0007 (7)
C19	0.0250 (9)	0.0317 (9)	0.0281 (8)	0.0001 (8)	−0.0049 (8)	−0.0012 (7)
C20	0.0198 (8)	0.0335 (9)	0.0342 (9)	0.0021 (7)	−0.0047 (8)	−0.0093 (7)
C21	0.0220 (8)	0.0333 (9)	0.0290 (8)	−0.0032 (8)	0.0025 (7)	−0.0103 (7)
C22	0.0236 (8)	0.0272 (8)	0.0226 (7)	−0.0062 (7)	0.0012 (7)	−0.0042 (7)
C23	0.0293 (9)	0.0276 (8)	0.0246 (8)	−0.0039 (8)	0.0021 (7)	−0.0001 (7)
O3	0.0383 (8)	0.0247 (6)	0.0348 (7)	0.0054 (6)	0.0101 (6)	0.0022 (5)
O4	0.0343 (7)	0.0258 (6)	0.0319 (6)	0.0045 (6)	0.0064 (6)	0.0018 (5)
N3	0.0281 (8)	0.0326 (8)	0.0309 (8)	−0.0022 (7)	0.0067 (7)	0.0051 (6)
N4	0.0237 (8)	0.0375 (8)	0.0319 (8)	0.0004 (7)	0.0052 (7)	−0.0076 (7)
C24	0.0258 (9)	0.0273 (8)	0.0241 (8)	−0.0027 (7)	−0.0007 (7)	−0.0012 (7)
C25	0.0236 (9)	0.0357 (9)	0.0315 (9)	0.0000 (8)	−0.0001 (8)	−0.0055 (8)
C26	0.0294 (9)	0.0293 (9)	0.0333 (9)	0.0058 (8)	−0.0074 (8)	−0.0061 (7)
C27	0.0339 (10)	0.0250 (8)	0.0284 (8)	0.0005 (8)	−0.0060 (8)	0.0009 (7)
C28	0.0275 (9)	0.0251 (8)	0.0256 (8)	−0.0015 (7)	−0.0012 (7)	0.0018 (7)
C29	0.0221 (8)	0.0249 (7)	0.0203 (7)	−0.0025 (7)	−0.0028 (7)	−0.0017 (6)
C30	0.0267 (9)	0.0229 (7)	0.0232 (8)	−0.0021 (7)	−0.0004 (7)	−0.0008 (6)
C31	0.0306 (9)	0.0255 (8)	0.0282 (8)	−0.0019 (8)	0.0017 (8)	0.0027 (7)
C32	0.0273 (9)	0.0232 (7)	0.0237 (8)	−0.0002 (7)	−0.0010 (7)	−0.0009 (6)
C33	0.0266 (9)	0.0237 (7)	0.0248 (8)	0.0014 (7)	0.0007 (7)	−0.0020 (6)
C34	0.0272 (8)	0.0234 (7)	0.0210 (7)	0.0006 (7)	−0.0020 (7)	−0.0008 (6)
C35	0.0280 (9)	0.0231 (7)	0.0237 (8)	0.0019 (7)	0.0008 (7)	−0.0005 (6)
C36	0.0240 (8)	0.0266 (8)	0.0223 (7)	0.0000 (7)	−0.0028 (7)	−0.0014 (6)
C37	0.0245 (8)	0.0257 (8)	0.0240 (8)	−0.0002 (7)	−0.0010 (7)	−0.0012 (6)
C38	0.0235 (8)	0.0260 (8)	0.0263 (8)	−0.0012 (7)	−0.0022 (7)	−0.0029 (7)
C39	0.0203 (8)	0.0255 (8)	0.0271 (8)	−0.0011 (7)	−0.0009 (7)	−0.0060 (7)
C40	0.0208 (8)	0.0275 (8)	0.0228 (8)	−0.0021 (7)	0.0015 (7)	−0.0058 (7)
C41	0.0256 (9)	0.0283 (8)	0.0272 (8)	−0.0014 (7)	0.0028 (7)	−0.0044 (7)
C42	0.0319 (10)	0.0282 (8)	0.0319 (9)	−0.0001 (8)	−0.0002 (8)	−0.0012 (7)
C43	0.0387 (11)	0.0341 (9)	0.0275 (8)	−0.0070 (9)	0.0017 (9)	0.0023 (7)
C44	0.0310 (10)	0.0416 (10)	0.0247 (8)	−0.0064 (9)	0.0048 (8)	−0.0029 (8)
C45	0.0224 (8)	0.0321 (9)	0.0258 (8)	−0.0025 (8)	0.0011 (7)	−0.0077 (7)
C46	0.0234 (8)	0.0319 (8)	0.0281 (8)	−0.0001 (8)	−0.0015 (7)	−0.0054 (7)
O5	0.0364 (8)	0.0346 (7)	0.0295 (6)	0.0036 (6)	−0.0067 (6)	−0.0032 (5)

### Geometric parameters (Å, °)

**Table:**

O1—C11	1.331 (2)	O3—H3O	0.8981
O1—H1O	0.9805	O4—C36	1.282 (2)
O2—C13	1.271 (2)	N3—C31	1.355 (2)
N1—C8	1.369 (2)	N3—C24	1.385 (2)
N1—C1	1.376 (2)	N3—H3N	0.9343
N1—H1N	0.8961	N4—C46	1.357 (2)
N2—C23	1.359 (2)	N4—C45	1.381 (2)
N2—C22	1.381 (2)	N4—H4N	0.9078
N2—H2N	0.8622	C24—C25	1.389 (3)
C1—C2	1.400 (2)	C24—C29	1.411 (2)
C1—C6	1.412 (2)	C25—C26	1.379 (3)
C2—C3	1.379 (3)	C25—H25	0.9500
C2—H2	0.9500	C26—C27	1.401 (3)
C3—C4	1.405 (3)	C26—H26	0.9500
C3—H3	0.9500	C27—C28	1.379 (2)
C4—C5	1.384 (2)	C27—H27	0.9500
C4—H4	0.9500	C28—C29	1.407 (2)
C5—C6	1.402 (2)	C28—H28	0.9500
C5—H5	0.9500	C29—C30	1.449 (2)
C6—C7	1.449 (2)	C30—C31	1.380 (2)
C7—C8	1.384 (2)	C30—C32	1.432 (2)
C7—C9	1.446 (2)	C31—H31	0.9500
C8—H8	0.9500	C32—C33	1.348 (2)
C9—C10	1.341 (2)	C32—H32	0.9500
C9—H9	0.9500	C33—C34	1.438 (2)
C10—C11	1.454 (2)	C33—H33	0.9500
C10—H10	0.9500	C34—C35	1.383 (2)
C11—C12	1.375 (2)	C35—C36	1.413 (2)
C12—C13	1.433 (2)	C35—H35	0.9500
C12—H12	0.9500	C36—C37	1.453 (2)
C13—C14	1.458 (2)	C37—C38	1.350 (2)
C14—C15	1.349 (2)	C37—H37	0.9500
C14—H14	0.9500	C38—C39	1.434 (2)
C15—C16	1.435 (2)	C38—H38	0.9500
C15—H15	0.9500	C39—C46	1.387 (3)
C16—C23	1.389 (2)	C39—C40	1.445 (2)
C16—C17	1.446 (2)	C40—C41	1.400 (3)
C17—C18	1.409 (2)	C40—C45	1.414 (2)
C17—C22	1.413 (2)	C41—C42	1.383 (2)
C18—C19	1.383 (2)	C41—H41	0.9500
C18—H18	0.9500	C42—C43	1.409 (3)
C19—C20	1.403 (3)	C42—H42	0.9500
C19—H19	0.9500	C43—C44	1.381 (3)
C20—C21	1.382 (3)	C43—H43	0.9500
C20—H20	0.9500	C44—C45	1.393 (3)
C21—C22	1.395 (3)	C44—H44	0.9500
C21—H21	0.9500	C46—H46	0.9500
C23—H23	0.9500	O5—H5OA	0.8980
O3—C34	1.327 (2)	O5—H5OB	0.9121
			
C11—O1—H1O	100.4	C31—N3—C24	109.34 (15)
C8—N1—C1	109.19 (15)	C31—N3—H3N	127.8
C8—N1—H1N	124.9	C24—N3—H3N	122.9
C1—N1—H1N	125.5	C46—N4—C45	109.37 (16)
C23—N2—C22	108.92 (15)	C46—N4—H4N	125.2
C23—N2—H2N	121.8	C45—N4—H4N	125.1
C22—N2—H2N	129.2	N3—C24—C25	129.30 (17)
N1—C1—C2	128.77 (17)	N3—C24—C29	107.34 (16)
N1—C1—C6	108.13 (15)	C25—C24—C29	123.34 (16)
C2—C1—C6	123.07 (17)	C26—C25—C24	117.31 (18)
C3—C2—C1	117.15 (17)	C26—C25—H25	121.3
C3—C2—H2	121.4	C24—C25—H25	121.3
C1—C2—H2	121.4	C25—C26—C27	120.85 (17)
C2—C3—C4	121.07 (17)	C25—C26—H26	119.6
C2—C3—H3	119.5	C27—C26—H26	119.6
C4—C3—H3	119.5	C28—C27—C26	121.63 (17)
C5—C4—C3	121.40 (18)	C28—C27—H27	119.2
C5—C4—H4	119.3	C26—C27—H27	119.2
C3—C4—H4	119.3	C27—C28—C29	119.06 (17)
C4—C5—C6	119.15 (17)	C27—C28—H28	120.5
C4—C5—H5	120.4	C29—C28—H28	120.5
C6—C5—H5	120.4	C28—C29—C24	117.79 (16)
C5—C6—C1	118.15 (15)	C28—C29—C30	135.17 (16)
C5—C6—C7	135.34 (16)	C24—C29—C30	107.01 (15)
C1—C6—C7	106.51 (15)	C31—C30—C32	122.77 (16)
C8—C7—C9	122.09 (16)	C31—C30—C29	105.70 (16)
C8—C7—C6	106.21 (15)	C32—C30—C29	131.31 (16)
C9—C7—C6	131.69 (16)	N3—C31—C30	110.60 (16)
N1—C8—C7	109.95 (16)	N3—C31—H31	124.7
N1—C8—H8	125.0	C30—C31—H31	124.7
C7—C8—H8	125.0	C33—C32—C30	127.02 (16)
C10—C9—C7	127.92 (17)	C33—C32—H32	116.5
C10—C9—H9	116.0	C30—C32—H32	116.5
C7—C9—H9	116.0	C32—C33—C34	123.11 (16)
C9—C10—C11	122.78 (16)	C32—C33—H33	118.4
C9—C10—H10	118.6	C34—C33—H33	118.4
C11—C10—H10	118.6	O3—C34—C35	120.55 (17)
O1—C11—C12	121.28 (15)	O3—C34—C33	117.22 (16)
O1—C11—C10	117.16 (16)	C35—C34—C33	122.22 (15)
C12—C11—C10	121.56 (16)	C34—C35—C36	121.69 (16)
C11—C12—C13	121.16 (16)	C34—C35—H35	119.2
C11—C12—H12	119.4	C36—C35—H35	119.2
C13—C12—H12	119.4	O4—C36—C35	120.81 (16)
O2—C13—C12	120.52 (16)	O4—C36—C37	120.72 (16)
O2—C13—C14	121.95 (15)	C35—C36—C37	118.45 (15)
C12—C13—C14	117.54 (15)	C38—C37—C36	122.78 (16)
C15—C14—C13	123.55 (16)	C38—C37—H37	118.6
C15—C14—H14	118.2	C36—C37—H37	118.6
C13—C14—H14	118.2	C37—C38—C39	126.99 (17)
C14—C15—C16	126.76 (16)	C37—C38—H38	116.5
C14—C15—H15	116.6	C39—C38—H38	116.5
C16—C15—H15	116.6	C46—C39—C38	123.12 (17)
C23—C16—C15	123.09 (16)	C46—C39—C40	105.68 (16)
C23—C16—C17	105.58 (16)	C38—C39—C40	131.10 (17)
C15—C16—C17	131.27 (15)	C41—C40—C45	118.24 (16)
C18—C17—C22	117.73 (16)	C41—C40—C39	134.74 (16)
C18—C17—C16	135.35 (16)	C45—C40—C39	107.00 (16)
C22—C17—C16	106.91 (14)	C42—C41—C40	119.42 (17)
C19—C18—C17	119.33 (16)	C42—C41—H41	120.3
C19—C18—H18	120.3	C40—C41—H41	120.3
C17—C18—H18	120.3	C41—C42—C43	120.94 (19)
C18—C19—C20	121.51 (17)	C41—C42—H42	119.5
C18—C19—H19	119.2	C43—C42—H42	119.5
C20—C19—H19	119.2	C44—C43—C42	121.10 (17)
C21—C20—C19	120.75 (17)	C44—C43—H43	119.4
C21—C20—H20	119.6	C42—C43—H43	119.4
C19—C20—H20	119.6	C43—C44—C45	117.35 (18)
C20—C21—C22	117.53 (17)	C43—C44—H44	121.3
C20—C21—H21	121.2	C45—C44—H44	121.3
C22—C21—H21	121.2	N4—C45—C44	129.63 (17)
N2—C22—C21	128.92 (16)	N4—C45—C40	107.52 (16)
N2—C22—C17	107.94 (16)	C44—C45—C40	122.85 (18)
C21—C22—C17	123.12 (16)	N4—C46—C39	110.41 (17)
N2—C23—C16	110.64 (16)	N4—C46—H46	124.8
N2—C23—H23	124.7	C39—C46—H46	124.8
C16—C23—H23	124.7	H5OA—O5—H5OB	100.6
C34—O3—H3O	108.8		
			
C7—C8—C9—C10	0.8 (3)	C30—C31—C32—C33	5.6 (3)
C8—C9—C10—C11	178.9 (2)	C31—C32—C33—C34	174.1 (2)
C9—C10—C11—C12	176.78 (16)	C32—C33—C34—C35	176.24 (18)
C10—C11—C12—C13	−177.21 (16)	C33—C34—C35—C36	179.28 (16)
C11—C12—C13—C14	176.22 (15)	C34—C35—C36—C37	−177.59 (16)
C12—C13—C14—C15	−169.74 (17)	C35—C36—C37—C38	−172.36 (16)
C13—C14—C15—C16	−179.53 (16)	C36—C37—C38—C39	−174.38 (17)

### Hydrogen-bond geometry (Å, °)

**Table:**

*D*—H···*A*	*D*—H	H···*A*	*D*···*A*	*D*—H···*A*
O1—H1O···O2	0.98	1.58	2.5148 (18)	158
N1—H1N···N1^i^	0.90	2.55	3.4254 (18)	167
N2—H2N···O5^ii^	0.86	2.08	2.912 (2)	161
O3—H3O···O4	0.90	1.71	2.5120 (19)	147
O5—H5OA···O5^iii^	0.90	2.11	3.0062 (10)	177
O5—H5OB···O4^iv^	0.91	1.83	2.7128 (18)	162

Symmetry codes: (i) *x*+1/2, −*y*+3/2, −*z*; (ii) *x*−3/2, −*y*+3/2, −*z*+1; (iii) *x*−1/2, −*y*+3/2, −*z*+1; (iv) *x*+1, *y*, *z*.
